# Dr. Paine’s Discoveries

**Published:** 1887-11

**Authors:** 


### DR. PAINE'S DISCOVERIES.

We call attention to the letter from Dr. F. T. Paine, of Comanche, published in our October number. Since the date of that letter we have received another, containing further information on the subject; and believing that it will prove of interest to our readers we refer to it here.

Under date of October 20, the doctor writes: "Satisfied that I am elucidating some of the great problems of the pathology of the nervous system, I am more than anxious at my time of life [the Dr. is 73 years of age] to have them verified; possibly I may enlist some abler man with better facilities to carry out what I have only begun; I am yet only a student.

I had an opportunity yesterday to establish to my own satisfaction, a theory I have long entertained, viz: that impressions made upon the peripheral terminations of the different nerves by the Farradic current, are entirely different, diametrically opposite—they are the antitheses, the antipodes, (so to speak), the antagonists, *and the antidote* to all painful impressions, made by any other means whatever!

My daughter, while preparing breakfast, scalded her wrist so as to take off the epidermis. The only application was the Farradic current, through a water bath, powerful enough to contract the muscles most violently, and the pain of the burn was INSTANTLY RELIEVED, her cooking was resumed, and breakfast was delayed only thirty minutes. It is now thirty-six hours since the scald, and although the skin is removed, she has not had a moments pain.

The interest is not in this single case, but in the rationale; how is the pain relieved by, what the Erb calls "the irritant *par excellence.*"

I am aware that all I have published is enigmatical and paradoxical to the profession and to the laity. I only need an opportunity to make it plain."

Dr. Paine should be furnished with the facilities to make the proposed demonstration; and, in this connection we are authorized to say he proposes to visit the Austin District Medical Society for the purposes set forth in his letter, at his own expense—some four hundred miles; but, while offering to visit other societies for the same purposes, it is but reasonable and fair that the society should take his visit as a compliment, and common politeness would suggest that it be without expense to him. In our judgment Dr. Paine has struck a lead that will doubtless open up a rich mine, a field worthy the cultivation of the ablest scientists, and that his name will go down the "corridors of time" along with that of the great electricians of the world. So mote it be.

The East Line Medical Association have extended an invitation to Dr. Paine to attend their next meeting, and demonstrate his theories and observations.

P. S. Just as the November number goes to press we learn with regret that the venerable doctor is suffering with paralysis of the right arm; no cause assigned. He writes, however, that he still hopes to be able to meet with the Austin District Medical Association, December 8. In a p. s. Dr. Paine says "I am now treating burns and scalds with electricity alone, and the pain is *iustantly* relieved.

